# Cross-Sectional Imaging Instead of Colonoscopy in Inflammatory Bowel Diseases: Lights and Shadows

**DOI:** 10.3390/jcm11020353

**Published:** 2022-01-12

**Authors:** Ludovico Alfarone, Arianna Dal Buono, Vincenzo Craviotto, Alessandra Zilli, Gionata Fiorino, Federica Furfaro, Ferdinando D’Amico, Silvio Danese, Mariangela Allocca

**Affiliations:** 1 Division of Gastroenterology and Digestive Endoscopy, Department of Gastroenterology, IRCCS Humanitas Research Hospital, Via Manzoni 56, 20089 Rozzano, MI, Italy; ludovico.alfarone@humanitas.it (L.A.); arianna.dalbuono@humanitas.it (A.D.B.); vincenzo.craviotto@humanitas.it (V.C.); federica.furfaro@humanitas.it (F.F.); 2 Gastroenterology and Endoscopy, IRCCS Hospital San Raffaele, University Vita-Salute San Raffaele, 20132 Milan, MI, Italy; zilli.alessandra@hsr.it (A.Z.); fiorino.gionata@hsr.it (G.F.); damico.ferdinando@hsr.it (F.D.); sdanese@hotmail.com (S.D.); 3 Department of Biomedical Sciences, Humanitas University, Via Rita Levi Montalcini 4, 20090 Pieve Emanuele, MI, Italy

**Keywords:** bowel ultrasound, inflammatory bowel disease, cross-sectional imaging, transmural healing, point-of care

## Abstract

International guidelines recommend a treat-to-target strategy with a close monitoring of disease activity and therapeutic response in inflammatory bowel diseases (IBD). Colonoscopy (CS) represents the current first-line procedure for evaluating disease activity in IBD. However, as it is expensive, invasive and poorly accepted by patients, CS is not appropriate for frequent and repetitive reassessments of disease activity. Recently, cross-sectional imaging techniques have been increasingly shown as reliable tools for assessing IBD activity. While computed tomography (CT) is hampered by radiation risks, routine implementation of magnetic resonance enterography (MRE) for close monitoring is limited by its costs, low availability and long examination time. Novel magnetic resonance imaging (MRI)-based techniques, such as diffusion-weighted imaging (DWI), can overcome some of these weaknesses and have been shown as valuable options for IBD monitoring. Bowel ultrasound (BUS) is a noninvasive, highly available, cheap, and well accepted procedure that has been demonstrated to be as accurate as CS and MRE for assessing and monitoring disease activity in IBD. Furthermore, as BUS can be quickly performed at the point-of-care, it allows for real-time clinical decision making. This review summarizes the current evidence on the use of cross-sectional imaging techniques as cost-effective, noninvasive and reliable alternatives to CS for monitoring patients with IBD.

## 1. Introduction

Inflammatory bowel diseases (IBD), such as Crohn’s disease (CD) and ulcerative colitis (UC), are chronic, recurrent and progressive inflammatory gastrointestinal disorders that can lead to invalidating complications [1,2]. Therapeutic strategies aiming merely at controlling symptoms have been demonstrated to fail in modifying the natural course of these diseases. Thus, there has been a shift towards a treat-to-target approach based on a tight control of the disease activity with close monitoring of intestinal inflammation through objective interval assessments and therapy optimization whenever the therapeutic targets are not met [3]. This strategy has been proven able to impact on the disease course, improving long-term outcomes in IBD patients [4]. Colonoscopy (CS) is the gold standard for assessing disease activity and severity in IBD [5,6]. Nevertheless, CS cannot entirely assess and quantify the small bowel disease extension and detect any transmural and extramural CD activity, including complications such as fistulas and abscesses [7]. In addition, it is an expensive and invasive procedure with the risk, although low, of bowel perforation, and its repetition over time is poorly tolerated by patients [8,9]. These limitations make CS an unsuitable tool for the frequent and repetitive monitoring required by the treat-to-target strategy. Hence, noninvasive, cost-effective and easy-to-use options are strongly needed for the routine care.

In the last few years, the use of cross-sectional imaging techniques for IBD assessment has significantly grown. Computed tomography enterography (CTE) has been proven to have high accuracy for the detection of small bowel disease extension and complications in CD, but its use is limited by radiation exposure [10,11]. Magnetic resonance enterography (MRE) is currently the recommended procedure for evaluating the small bowel and complications in CD, and it has been suggested as a potential alternative to CS for the assessment of both UC and ileo-colonic CD [6,12,13]. However, standard MRE is costly, time-consuming and not promptly accessible; in addition, it requires bowel preparation and the intravenous injection of gadolinium as a contrast agent, which carries the risk of adverse events such as allergic reactions and nephrogenic systemic fibrosis, and, therefore, it can be scarcely accepted by patients [14]. New magnetic resonance imaging (MRI)-based techniques can represent an added value in the monitoring of IBD, overcoming some limitations of conventional MRE and providing further information. Among these, diffusion-weighted imaging (DWI) has demonstrated good accuracy and reliability, but lacks an adequate standardization of DWI scanners and procedures [15]. In addition to CT and MRE, bowel ultrasound (BUS) is an inexpensive, noninvasive, readily available and well tolerated procedure that does not require either bowel preparation or contrast agent and can be performed at the point-of-care [16]. It is as valuable as CS and MRE for detecting disease activity and complications in IBD [17]. The aim of this review is to discuss the use of cross-sectional imaging techniques as less invasive, cost-effective and accurate substitutes of CS in the management of IBD with a particular focus on those clinical scenarios where they might perform superiorly to endoscopy.

## 2. Crohn’s Disease

### 2.1. Cross-Sectional Imaging Techniques in Crohn’s Disease

In CD, the inflammation typically develops transmurally, resulting in destructive complications such as strictures, fistulas, and abscesses, requiring surgery over time in about half of patients [1]. Cross-sectional imaging techniques, including CTE, MRE and BUS, are required to assess and monitor the entire disease burden, including the small bowel and transmural locations, as indicated by the European Crohn’s and Colitis Organization (ECCO) and the European Society of Gastrointestinal and Abdominal Radiology (ESGAR) latest guidelines [6,17].

### 2.2. Computed Tomography Enterography for the Assessment of Disease Activity and Complications

Several data are available on the accuracy of CTE in assessing disease activity and complications in CD. In a systematic review by Panes et al., according to the high-quality standards required, 69 relevant prospective studies were included [10]. CTE was demonstrated as a valuable tool for assessing CD activity (overall sensitivity and specificity of 81% and 88%, respectively) and for detecting stricturing and/or penetrating complications (sensitivity and specificity higher than 80%) [10]. Furthermore, a meta-analysis performed by Horsthuis et al. showed that CTE has high accuracy in detecting CD (mean per-patient sensitivity and specificity were 84% and 95%, respectively; mean per-bowel-segment sensitivity and specificity were 68% and 90%, respectively) [18]. The same authors in a later meta-analysis evaluating the accuracy of cross-sectional imaging techniques in grading CD activity compared to CS showed that CTE accurately classified CD activity (per-patient and per segment accuracy grading were 95% and 87%, respectively) [19]. CTE, compared with MRI techniques, is quicker, cheaper and more available [20]. There are, however, a few limitations of this imaging technique: it requires the ingestion of 1500–2000 mL of oral contrast to achieve adequate small bowel distension and the intravenous administration of an iodine contrast agent, which impairs tolerability [21]. Additionally, the exposure to ionizing radiations, leading to increased cancer risk (16% of CD patients have been shown to have an increased cancer risk of 7% due to radiation use), prevents the application of CTE for a regular monitoring of CD over time [11,22]. Thus, the utilization of CTE is usually confined to emergencies that often require a surgical evaluation. Of note, guidelines suggest the use of CTE as a first-line procedure whenever an intra-abdominal abscess is suspected [6].

### 2.3. Magnetic Resonance Enterography

#### 2.3.1. Assessment of Disease Activity and Complications

Horsthuis et al. in their meta-analyses reported MRE as reliable both for the diagnosis of suspected CD (both mean per-patient sensitivity and specificity were 93%; mean per-bowel-segment sensitivity and specificity were 70% and 94%, respectively) and for grading CD activity (per-patient and per segment accuracy grading were 84% and 67–82%, respectively) [18,19].

Moreover, MRE has been demonstrated to be accurate for assessing CD activity (sensitivity 80% and specificity 82%) and for identifying fistulas, abscesses and strictures (sensitivity and specificity were 76% and 96%, 86% and 93%, and 89% and 94%, respectively) as reported by Panes et al. in their systematic review [10]. Unlike CTE, MRE is a radiation-free procedure, suitable for frequent restaging and monitoring young CD patients over the time. Hence, it currently represents the gold standard investigation for assessing small bowel CD presence and extension as well as transmural and extramural characteristics and complications [6]. Furthermore, MRE has a shorter recovery time and is considered more acceptable by CD patients in comparison to CS (88% vs. 60%, *p* < 0.001) [14]. As concerns the grading of activity and severity, Rimola et al. developed the Magnetic Resonance Index of Activity (MaRIA score), validated using CS as a reference standard, for an objective assessment of disease activity, combining several radiological features: bowel wall thickness (BWT), relative contrast enhancement, edema and ulcerations [12]. The global MaRIA score is obtained from the sum of the segmental MaRIA scores in the ileum, ascending colon, transverse colon, descending colon, sigmoid and rectum [12]. Both the segmental and the global MaRIA scores significantly correlated with the segmental and global Crohn’s Disease Endoscopic Index of Severity (CDEIS) score (*r* = 0.81, *p* < 0.001 and *r* = 0.78, *p* < 0.001), respectively [12]. The MaRIA score has proven to be a reliable tool for detecting ileo-colonic CD activity (the area under the receiver operating characteristic (AUROC) was 0.891) [12]. In an external validation study, Rimola et al. reported that BWT, relative contrast enhancement, edema and ulcerations detected on MRI independently predicted CD activity. In addition, they found a MaRIA score ≥ 7 and ≥11 accurately identified active and severe ileo-colonic CD, respectively (AUROC 0.93, sensitivity 87%, specificity 87% and AUROC 0.93, sensitivity 87%, specificity 87%, respectively) [23]. Finally, the MaRIA score has been proved to be reproducible and to have a good inter-observer variability (the intra-class correlation coefficients (ICC) were 0.70, *p* < 0.001) [24].

Although optimal MRE colonic sequences and adequate colonic distension are lacking, MRE was also proved able to identify colonic alterations in CD patients. Indeed, a meta-analysis by Chavoshi et al. found MRE detected colonic involvement in CD with a high pooled specificity (95%); the pooled sensitivity, positive and negative likelihood ratio was 69%, 14 and 0.31, respectively [25]. These results suggest that MRE is valuable to confirm colonic CD even if it is not accurate enough to exclude it as a single diagnostic investigation [25]. Of note, while specificity did not differ between adults and pediatrics, the sensitivity of MRE was higher in the pediatric population (80% vs. 62%) [25]; based on this evidence, MRE can be suggested as a first-line procedure for assessing colonic CD in pediatrics [25].

In summary, although the role of MRE in CD has conventionally been limited to evaluate small bowel alterations and transmural and extramural disease, these findings underscore MRE, through the use of the MaRIA score, which reliably evaluates activity, severity and complications in ileo-colonic CD.

#### 2.3.2. Monitoring and Prediction of Outcomes

A prospective study evaluated the accuracy of MRE in monitoring treatment responses in CD [26]. MRE was shown to identify ulcer healing (MaRIA score < 11) and mucosal healing (MH) (MaRIA score < 7) with 83% and 90% of accuracy, respectively [26]. Indeed, the MaRIA score accurately predicted ulcer healing and MH in response to therapy (AUROC 0.833, sensitivity 75%, specificity 80% and AUROC 0.864, sensitivity 83%, specificity 84%, respectively) [26]. The MaRIA score reliably detected changes in ulcer severity (Guyatt’s index was 1.2) [26]. Additionally, the extent of changes in MaRIA score and the CDEIS were well correlated with statistical significance (*r* = 0.51; *p* < 0.001) [26]. In a further comparison study between MRE and CS accuracy and impact on CD management, Garcìa-Bosch et al. assessed approaches adopting CS and MRE alternatively as first- and second-line investigations, analyzing clinical, endoscopic and radiological data, including therapeutic decisions, from 100 patients with ileo-colonic CD [27]. The use of MRE as a first examination was shown sufficient for changing the therapeutic management in 80% of CD cases, while this occurred only in 34% of CD patients when CS was the first investigation (*p* < 0.001) [27]; when used as a second examination, MRE changed therapeutic management more frequently than CS (28% vs. 8%, *p* < 0.001) [27]. As concerns long-term outcomes, Buisson et al. showed that transmural healing (TH) assessed by MRE predicted sustained clinical corticosteroid-free remission (odds ratio (OR) 4.42, *p* = 0.042) and decreased the risk of CD-related surgery (hazard ratio (HR) 0.16, *p* = 0.008) [28]. Another study by Fernandes et al. reported that patients with TH assessed by MRE had lower hospital admission rates, therapy escalation and surgery than those with MH or no healing [29].

Overall, MRE has been shown as sensitive to therapeutic response in CD patients. Indeed, MaRIA score accurately detects endoscopic response and remission. Furthermore, TH assessed by MRE may have an added value in predicting long-term outcomes in comparison to MH assessed by CS.

Thus, MRE could represent an attractive, less invasive and more acceptable option than CS for close monitoring of patients with ileo-colonic CD over time. Nevertheless, MRE is an invasive, time-consuming, expensive and poorly available procedure requiring bowel preparation and the intravenous administration of gadolinium-based contrast agent; therefore, its widespread use is limited.

### 2.4. Diffusion Weighted Imaging

#### 2.4.1. Assessing Disease Activity

Diffusion-weighted imaging (DWI) is a magnetic resonance (MR)-based technique that analyzes water molecules’ motion in the extracellular and intracellular compartments to provide image contrast. It can be performed without the need for intravenous administration of gadolinium-based contrast agent. Due to restricted microscopic diffusion of water molecules, a high enhancement diffusion signal and a low apparent diffusion coefficient (ADC) value have been detected in inflamed bowel segments in IBD patients and allowed the identification of active CD [15]. A systematic review and meta-analysis by Choi et al. found DWI to be very accurate in evaluating CD activity: the pooled sensitivity and specificity were 92.9% and 91%, respectively [30].

In order to provide an objective and quantitative assessment of disease activity, the Nancy score for DWI imaging was developed. This score comprehends standard and DW-MRI radiological features: ulceration, parietal edema, BWT, differentiation between submucosa or mucosa and muscularis propria, rapid contrast enhancement, and DWI hyperintensity [31]. These radiological findings are dichotomously assessed (0/1), and the sum of the values taken from each of the bowel segments (rectum, sigmoid colon, left colon, transverse colon, right colon, and ileum) allows a total score to be estimated [31]. The Nancy score was validated in a cohort of 40 CD patients using CS as the reference standard [31]. Endoscopic inflammation (namely, evidence of erythema, edema, pseudopolyps, aphthoid ulcers, and ulcerations) was identified by a segmental Nancy score higher than 2 (AUROC 0.779; sensitivity 58%, specificity 84%; *p* = 0.0001) [31]. In addition, DWI hyperintensity accurately detected endoscopic inflammation (OR 2.67; AUROC 0.702; *p* = 0.0001). Furthermore, after the intravenous administration of gadolinium-based contrast agent, DWI hyperintensity and rapid contrast enhancement did not significantly differ in accuracy for identifying endoscopic inflammation (*p* = 0.58) [31]; thus, DWI sequences may efficiently substitute the intravenous contrast agent in this setting. Additionally, the total Nancy score was shown to be correlated with the Simplified Endoscopic Activity Score for CD (SES-CD) (*r* = 0.539; *p* = 0.001) [31].

DWI hyperintensity was demonstrated to highly correlate with the MaRIA score and reliably detected ileal activity (specificity 100%, sensitivity 93%) [32,33]. Moreover, ADC was found to be inversely correlated with the MaRIA score (*r* = −0.77, *p* = 0.0001) and a threshold ADC value of 1.6 × 10^−3^ mm^2^/s was distinguished as active from quiescent disease with a sensitivity of 82% and a specificity of 100% (AUROC, 0.96) [32,33]. The authors developed the Clermont index, a new score including three standard MRE features (BWT, edema, and ulceration) and replacing the relative contrast enhancement with ADC values [32,33]. The MaRIA score, used as a reference standard, and the Clermont index highly correlated in assessing and detecting ileal CD (*r* = 0.99) [32,33]. Clermont index scores > 8.4 and ≥12.5 were reported to predict active ileal CD, (MaRIA score ≥ 7) and severe ileal disease (MaRIA score ≥ 11), respectively (AUROC 0.99, *p* = 0.0001) [32,33]. In addition, a good agreement was demonstrated between the Clermont index and ileal CDEIS and SES-CD (*r* = 0.63, *p* < 0.05; *r* = 0.58, *p* < 0.05, respectively) [34]. In a prospective study using CS as the reference standard for assessing the diagnostic accuracy, the Clermont index was shown to accurately identify active CD (sensitivity 89%, specificity 78%, AUROC 0.84) and severe active disease (sensitivity 83%, specificity 89%, AUROC 0.86) [27]. Nevertheless, although a good inter-reader agreement in the assessment of ADC values was reported (ICC 0.918), the documented difference in ADC scanning techniques and parameters, as well as the relevant inter- and intra-scanner variability, significantly affect ADC values’ reliability and appear to prevent the implementation of ADC-based scores in clinical practice [35,36].

In summary, the exposed data show DWI to be reliable in assessing disease activity in CD. In particular, the Nancy score has been found to be simple, reproducible and well correlated with SES-CD.

#### 2.4.2. Monitoring Patients

Thierry et al. conducted a prospective study on 96 CD patients undergoing DWI-MR before and after biological therapy [37]; at the same time, CS was also performed in a subgroup of 20 patients [37]. The total and the segmental Nancy score significantly correlated with the total and the segmental CDEIS (*r* = 0.60, *p* < 0.0001; and *r* = 0.63, *p* < 0.0001, respectively) [37]. A total Nancy score lower than 6 and a segmental Nancy score lower than 2 reliably detected both the total (sensitivity, specificity, and accuracy were 70%, 80%, and 75%, respectively (AUROC, 0.82; *p* < 0.0001)), and the segmental endoscopic MH (sensitivity, specificity and accuracy were 92%, 68%, and 85%, respectively (AUROC, 0.80; *p* < 0.0001)) [37]. Both the total and the segmental Nancy score demonstrated good intra-rater reliability and inter-rater reliability (ICC were greater than 0.90 for both, *p* < 0.0001) [37]. Moreover, both the total and the segmental Nancy score were found to be sensitive to treatment responses (Guyatt’s responsiveness index was 1.18 and 0.85, respectively) [37]. Finally, DW-MRI identification of MH was correlated with a lower risk of intestinal resection (*p* < 0.05) [37].

Overall, these findings demonstrate DWI can accurately identify therapeutic response in CD patients and may have a role in predicting CD course. Compared to standard MRE, DWI is faster, easier, less invasive and more tolerable (intravenous contrast agent is not required and neither fasting nor bowel preparation is needed for colonic evaluation). However, it is inferior to MRE for the assessment of non-perianal penetrating complications due to a less detailed anatomic visualization [38]. Furthermore, the lack of standardization of DWI scanners and examinations makes DWI sequences heterogeneous [39].

### 2.5. Other New Magnetic Resonance Imaging-Based Techniques

Magnetization transfer (MT) is a novel MRI technique that provides contrast between protons in free water molecules and protons in large macromolecules, such as collagen, without the administration of any intravenous contrast agent. The MT signal of a specific tissue can be quantified by the MT ratio, which increases along with the increase in collagen in that tissue. MT-based MR may, therefore, estimate the relative amount of fibrosis in a tissue [40]. The development of bowel wall strictures occurs in about 30% of CD patients and this complication requires medical, endoscopic or surgical therapy depending on the nature of the stricture. However, while strictures can be accurately diagnosed by both CS and cross-sectional imaging techniques (BUS, CTE, MRE), no diagnostic investigation has been proved efficient to discriminate the nature of the stricture (mainly fibrotic or mainly inflammatory) and to establish the degree of fibrosis in the bowel wall [10,41]. Li et al. compared contrast-enhanced MRE, DWI, and MT imaging in determining the degree of fibrosis in 31 CD patients with small-bowel strictures, using surgical histopathologic analysis as a reference standard [42]. The MT ratio highly correlated with fibrosis scores (*r* = 0.769) but not with inflammation scores (*r* = −0.034) and differentiated nonfibrotic from fibrotic bowel segments more accurately than ADC and percentage of contrast enhancement gain (AUROC 0.981 vs. 0.869 and 0.646, respectively) [42]. In addition, the MT ratio was more reliable in distinguishing moderate-to-severe fibrosis from mild-to-absent fibrosis than ADC and percentage of contrast enhancement gain (AUROC 0.919 vs. 0.747 and 0.592, respectively) [42]. A recent study carried out by Fang et al. confirmed these findings, showing that MT ratio significantly correlated with histological fibrosis scores (*r* = 0.681, *p* < 0.001) and was more sensitive and specific in discriminating mild from moderate-to-severe fibrosis than contrast-enhanced MRE (sensitivity and a specificity of 0.913 and 0.923 vs. 0.871 and 0.800, respectively) [43].

Another new MRI technique is motility MRI (mMRI), whose sequences can quickly capture the same segment of bowel over time, providing dynamic images. Indeed, mMRI gives information about small bowel function and motility, which cannot be obtained through CS and conventional cross-sectional imaging techniques. A study on the application of mMRI in a small cohort of CD patients found that the assessed small bowel contraction frequency agreed with calprotectin (*r* = −0.85) and CRP (*r* = −0.701) [44]. Recently, Menys et al. reported a correlation between terminal ileal mMRI-measured motility and both CDEIS and endoscopic acute histologic inflammatory score (EAIS) (AUROC 0.86, *r* = −0.59 and AUROC 0.87, *r* = −0.61, respectively) [45]. Moreover, MRI-assessed motility was found to increase in anti-TNF responders than non-responders (median increase of 73.4% vs. median reduction of 25%, *p* < 0.001); furthermore, MRI-measured motility accurately identified the response to anti-TNF therapy (sensitivity 93.1%, specificity 76.5%) [46]. In summary, MT-based MR and mMRI are novel promising tools, which can provide additional data on CD patients, not obtainable from CS and conventional cross-sectional imaging techniques. Of note, the ability of the MT ratio to discriminate the nature of small bowel strictures could potentially have a huge impact on CD management; mMRI-measured small bowel motility could be used for assessing activity and therapeutic response. Further larger prospective studies are awaited to confirm these findings.

### 2.6. Bowel Ultrasound

#### 2.6.1. Assessment of Disease Activity and Complications

The comparison of BUS with different imaging techniques for accuracy in assessing CD patients has been carried out by systematic reviews and meta-analyses [10,18,19,47,48]. The overall per-patient sensitivity and specificity of BUS for the diagnosis of CD were 85% and 98%, compared with 93% and 93% for MRE, respectively [10,18,19,47,48]. The sensitivity and specificity of BUS for the detection of activity were 85% and 91% against 81% and 88% for CTE and 80% and 82% for MRE [10,18,19,47,48]. Furthermore, the sensitivity and specificity of BUS for the assessment of fistulas, abscesses, and strictures were 74% and 95%, 84% and 93%, and 79% and 92%, while MRE’s sensitivity and specificity were 76% and 96%, 86% and 93%, and 89% and 94%, respectively [10,18,19,47,48].

Recently, Rispo et al. demonstrated a high correlation between BUS and MRE (*r* = 0.9; *p* < 0.001) for the assessment of bowel damage in CD patients with the use of the Lémann index [49]. A prospective multicenter clinical trial evaluated the diagnostic accuracy of BUS and MRE for the presence, extent, and activity of CD in a large cohort of CD patients [50]. Both BUS and MRE had an accuracy > 90% for individuating small bowel CD. MRE had significantly higher sensitivity and specificity than BUS (10% and 14% difference for extent and 5% and 12% for presence) [50]. However, BUS was not performed by gastroenterologists with expertise and skills in the use of BUS in IBD [50]. In another study, BUS accuracy in assessing CD’s activity and complications was evaluated using a combination of MRE and CS as reference standards [51]. BUS was found to be highly reliable in detecting CD localization (sensitivity and specificity were 88% and 96%, respectively), disease activity (sensitivity and specificity were 92% and 100%, respectively), strictures (sensitivity and specificity were 75% and 86%, respectively) and penetrating complications (sensitivity and specificity were 100% and more than 96%, respectively) [51]. Notably, in this study, BUS-driven management of CD patients correlated well (*r* = 0.768, *p* < 0.001) with clinicians’ decisions based on clinical parameters, biomarkers, CS and MRE findings [51]. A recent study on the relevance of intravenous contrast agent in the ultrasonographic assessment of CD activity demonstrated that the increase in parietal enhancement after contrast injection of ≥47% assessed by contrast-enhanced ultrasound (CEUS) and BWF > 1 evaluated by BUS had the same predictive positive value for identifying ulcers at CS (97% vs. 100%) [52]. Indeed, CEUS, together with color Doppler signal and elastography, belongs to the BUS-based ancillary techniques, which could extend the diagnostic power of BUS, especially in selected settings. According to current guidelines, CEUS should only be used to discriminate between abscess and inflammatory masses and to demarcate fluid cavities for eventual percutaneous drainage [14]. Nevertheless, CEUS has been investigated for other purposes, such as the characterization of strictures in CD [53]; a study performed on 25 patients with CD strictures showed a good correlation between the sonographic and pathology scores for both inflammation and fibrosis (*r* = 0.53 and *r* = 0.50, respectively) [53]. Elastography, used for stiffness measurement of various tissues, has been explored as a potential tool to measure fibrosis in patients with IBD, particularly in recognizing predominantly fibrotic strictures in CD; a systematic review by Pescatori et al. reported that elastography findings correlated well with the degree of fibrosis [54]. However, its wide application is limited by the absence of standardization, the presence of different elastography modalities (shear wave and strain ratio), and the poor reproducibility. Color Doppler US can also be useful for the characterization of strictures; the loss of stratification and the hypervascularization may be US features associated with predominantly inflammatory strictures [55]. The scientific community has been increasingly focusing on the implementation of objective, repeatable, validated and reliable BUS activity scores in clinical practice. However, a systematic review by Bots et al., assessing the quality and reliability of the available published BUS activity scores for evaluating disease activity compared to reference standards, showed that most of the BUS scores were built through a low-quality development methodology not adhering to the high standards and criteria required [56]. The simple ultrasound score for Crohn’s disease (SUS-CD) was recently validated using SES-CD as a reference standard [57]. SUS-CD, composed by BWT and color Doppler imaging signal (CDS), has proved a high correlation with SES-CD (*r* = 0.78, *p* < 0.001) with great accuracy in detecting endoscopic activity (AUROC 0.92) [57]. According to a recent consensus, four key BUS parameters of CD activity have been identified: BWT; bowel wall stratification (BWS); (CDS); and inflammatory mesenteric fat (i-fat) [58]. Inter-rater reliability was very high for BWT (ICC 0.96, *p* < 0.001) and moderate to substantial for the remaining variables (ICC 0.45–0.62, *p* < 0.001) [58]. Taken together, BWT, CDS, i-fat, and BWS correlated with the global disease activity assessed by VAS scale (*r* was 0.73, 0.85, 0.93 and 0.87, respectively; *p* < 0.0001) and were included in the final International Bowel Ultrasound Segmental Activity Score (IBUS-SAS), which had excellent reliability (ICC 0.97, *p* < 0.001) [58].

A recent meta-analysis by Sagami et al. showed that BUS had a significantly lower diagnostic accuracy in identifying inflammation in the rectum (sensitivity 74.5%, specificity 69%) in comparison to the other colonic tracts in IBD patients [59]. Indeed, the rectum, located deep in the pelvis, is not easily examined by US due to distance and adipose tissue, which can attenuate waves and hinder the visualization. However, rectal assessment is indispensable, rectal involvement being highly valuable for management strategy, outcomes and quality of life in CD patients [60]. To overcome this weakness, transperineal ultrasound (TPUS) is emerging as a complementary tool to achieve a better diagnostic accuracy in identifying active disease and complications in every ileo-colonic segment, including the rectum. TPUS is more accessible and less invasive than the endo-rectal US, being able to assess the distal rectum, anal canal, anal sphincters and perianal tissues. Maconi et al. performed a systematic review and meta-analysis, which found TPUS as an accurate diagnostic tool to detect and classify perianal fistulas (with a sensitivity of 98.3% and a specificity of 92.8%, respectively) as well as perianal abscesses (sensitivity of 86%) [61].

In summary, there is accumulating evidence that BUS is as reliable and accurate as more invasive and expensive diagnostic investigations (MRE and CS) for detecting CD location, activity, severity and complications. TPUS in combination with BUS has been emerging as more diagnostically accurate than BUS alone. Table 1 summarizes the accuracy of CTE, MRE, DWI, and BUS and their advantages and limitations in CD.

#### 2.6.2. Predicting Outcomes and Monitoring

According to a recent meta-analysis, bowel US has also proved to accurately detect post-surgical recurrence, with a pooled sensitivity and specificity of 94% and 84%, respectively [62]. In particular, a BWT value ≥ 5.5 mm had high sensitivity and specificity (84% and 98%) in predicting a severe post-surgical recurrence (defined as Rutgeerts score ≥ 3) [62]. Recently, a 12-month prospective, observational, two-phase study was carried out on the role of baseline BUS findings in predicting CD course [63]. Firstly, baseline BWT (per 1 mm increase: OR 2.11; *p* < 0.001) and the presence of BWF (OR 5.24; *p* < 0.001) were shown as independent predictors for a baseline SES-CD > 2 [63]. Additionally, the authors designed a bowel US score (BUSS) combining BWT and BWF to assess disease activity [63]. A BUS score higher than 3.52 and the presence of any CD complications, such as strictures, abscesses and fistulas, at baseline BUS were identified as independent predictors of negative outcome at 12 months (OR 6.97; *p* < 0.001 and OR 3.90; *p* = 0.021, respectively) [63]. Of note, a subgroup analysis showed BUS score was also significantly predictive of a negative course at 12 months in CD patients in endoscopic remission (*p* < 0.003) [63]. In addition, BUS score also correlated with SES-CD (*r* = 0.55; *p* < 0.001) [63]. Furthermore, a subsequent prospective observational study on active CD patients starting biologics and/or immunosuppressants, using SES-CD as a reference standard, demonstrated that BUS score is also sensitive to therapeutic response. Indeed, authors found BUS score significantly changed from baseline to re-evaluation in patients achieving endoscopic response (*p* < 0.001) and remission (*p* < 0.003); conversely, no significant change in BUS score was reported in patients not achieving endoscopic response or remission [64]. The routine performance of point-of-care BUS in CD patients was demonstrated to lead to changes in the clinical management plan in up to 60% of subjects [65]. A study enrolling 30 CD patients starting therapy with immunomodulators and/or anti-TNF antibodies and followed up for 12 months found a good agreement between BUS remission (defined by BWT of ≤3 mm, bowel wall flow (BWF) of 0, and parietal enhancement gain after contrast administration less than 46%) and endoscopic remission (CDEIS < 6) (*r* = 0.73; *p* < 0.001) [66]. Of note, 83% of CD patients, who achieved MH, also reached BUS remission. BUS predicted highly reliability for MH (accuracy 86%, AUROC of 0.87); in this study, a BWT < 3 mm was shown as the best independent predictor of MH (96%) [66].

A prospective multicenter study by Ripollés et al. recruiting 51 patients with active CD starting treatment with anti-TNF therapy showed that an ultrasonographic response at 12 weeks (defined by at least a 2 mm decrease in BWT, a one-grade decrease in BWF, 20% decrease in the mural enhancement after contrast injection and/or disappearance of extramural complications) was predictive of an ultrasonographic response at one year (*p* < 0.0001) [67]. CD patients who did not achieve an ultrasonographic response at one year had a worse CD course (namely, modification of therapy or need for surgery) in the following year in comparison to those reaching an ultrasonographic improvement (13/20 (65%) vs. 3/28 (11%), *p* = 0.0001) [67]. Lately, Zorzi et al. found that CD patients treated with anti-TNF therapy for at least 12 months achieving an ultrasonographic response (e.g., improvement of all lesions detected at baseline US) had a significantly lower risk of need for surgery, hospitalization and steroids (*p* < 0.0001, *p* = 0.003 and *p* = 0.0001, respectively) comparing to US-non-responders [68].

In addition, unlike CS, BUS allows transmural assessment of the bowel wall, which is a relevant added value since there is growing evidence that TH is emerging as a more accurate treatment target than MH alone [29,69]. Castiglione et al. found that CD patients achieving TH at BUS, defined by BWT ≤ 3 mm, plus MH after two years of biological therapy, were at lower risk of clinical flares, hospitalization and surgery in the following year (HR 0.87, *p* = 0.01, HR 0.88, *p* = 0.002 and HR 0.94, *p* = 0.008, respectively) than those patients achieving MH alone [70].

The TRUST study, a 1-year longitudinal multicenter study recruiting 234 patients with active CD starting treatment intensification, further assessed the value of BUS as a tool for monitoring CD activity and therapeutic response over time. CD patients underwent BUS at baseline (start of therapy) and at three, six and twelve months. BWT, bowel wall pattern (BWP), BWF, presence of mesenteric lymph nodes, mesenteric hypertrophy and strictures were found significantly improved at all intervals (*p* < 0.005) [71]. The most relevant modifications were identified at three months [71]. Ascites, fistulas and pre-stenotic dilatations achieved a significant improvement at 12 months (*p* < 0.05) [71]. A decrease in BWT and a reduction in C-reactive protein (CRP) levels at three months were significantly correlated (*p* ≤ 0.001) [71]. Furthermore, an Italian multicenter study assessed the value of BUS for tight control and monitoring of CD patients treated with different biological therapies, including anti-TNF, ustekinumab and vedolizumab [72]. Modifications of BUS parameters were evaluated at baseline and after three, six and twelve months of treatment [72]. BUS was shown able to individuate improvement and remission even after a few months from the start of biological therapies, and BUS-guided therapy escalation after three months led to lesion improvement in 41% of patients [72].

The ultrasound substudy of STARDUST, a phase IIIB randomized clinical trial on CD patients treated with ustekinumab, analyzing very early modifications in BUS findings in response to therapy and the agreement of BUS improvement and remission with endoscopic and clinical outcomes, is currently ongoing [73]. Preliminary results showed a significant improvement of BWT even by week 4 (*p* = 0.0002) [73]. BUS response (e.g., >25% decrease in BWT vs. baseline) and TH (defined by normalization of BWT, color Doppler signal, echo stratification and inflammatory mesenteric fat) rates at week 16 were 33.8% and 11.3%, respectively [73]. BUS response at week 4 was found moderately correlated with clinical remission, reduction in CRP, fecal calprotectin and SES-CD at week 16 [73].

Overall, the established data, cited above, showed BUS findings can have a big role in detecting post-surgical recurrence and therapeutic responses and in predicting outcome. As BUS is fast, easy, cheap, readily available at the point-of-care and well accepted, it is proving to be very useful for close and tight monitoring over time. In addition, in the last few years, BUS activity scores are being developed with the aim of making BUS objective, standardized and reproducible.

### 2.7. Artificial Intelligence

Artificial intelligence (AI) is a section of computer science aimed at the setting up of machines capable of mimicking human cognitive processes [74]. Machine learning, a subset of AI that uses algorithms trained from labeled training dataset in order to detect specific patterns, is becoming more and more widely used in medicine [74]. Indeed, this technology allows the development of computer-aided detection (CAD) systems able to recognize pathological features, providing quick, objective and accurate diagnostic assessments and decreasing clinicians’ workload [74]. In order to achieve these purposes, there is growing interest in the application of AI for cross-sectional imaging in IBD, especially in CD. Some recent studies have shown that machine learning-assisted image analysis of CTE and MRI is significantly correlated with that of experienced radiologists and accurate for assessing disease activity and strictures in small bowel CD [75,76]. Furthermore, these AI-based image interpretation systems could also provide a better disease quantification, which may contribute to the personalization of clinical management [75,76]. In addition, a very recent study performed by Yang et al. has found a machine learning-based ultrasound as able to significantly discriminate inflamed and not inflamed bowel segments [77].

Overall, these preliminary encouraging findings, despite being scarce, underscore the potential added value of AI use for cross-sectional imaging in IBD. However, larger prospective randomized trials are awaited in the near future to prove the effectiveness and safety of such an approach before its implementation in real-life clinical scenarios.

## 3. Ulcerative Colitis—Cross-Sectional Imaging Techniques in Ulcerative Colitis

In order to achieve proper disease control, in UC patients, tight and close monitoring of intestinal inflammation is also required [3]. Besides the improvement of symptoms, including rectal bleeding and loose stools, MH, defined by a Mayo endoscopic subscore of 0 to 1, is designated as the target of treatment [3]; indeed, the achievement of MH in UC has been found to be associated with long-term clinical remission and decreased need for surgery and corticosteroid therapy [4]. CS is currently the gold standard procedure for the assessment of MH in UC [5].

In recent years, the use of cross-sectional imaging techniques, including CTE, MRE and US, is increasingly rising in UC.

### 3.1. Computed Tomography Enterography and Magnetic Resonance Enterography for Assessing Disease Activity

CTE has been shown to have a moderate accuracy in assessing disease activity in UC [78]. A comparison study showed that CTE findings moderately correlated with UC severity (*r* = 0.612) defined by endoscopic evaluation [78]. Johnson et al. reported that CTE in UC patients detected colonic inflammation with an overall sensitivity of 74% [79]; of note, moderate and severe diseases were identified with a sensitivity of 93% [79]. Similar to what has been said for its role in CD, the use of CTE, despite reduced costs and high availability, is limited by radiation exposure, especially for monitoring young patients over the disease course. Thus, CTE is the technique of choice to detect acute complications, such as toxic megacolon, bowel perforation, intra-abdominal complications and post-operative leaks [39].

A prospective study on MRE’s diagnostic accuracy for the assessment of disease activity and severity in UC, using CS as the reference standard, showed that MRE has a high diagnostic accuracy in detecting endoscopic inflammation (sensitivity 87%, specificity 88%, AUROC 0.95; *p* < 0.001) and severe endoscopic lesions (sensitivity 83%, specificity 82%, AUROC 0.91; *p* < 0.001) [13].

In summary, CTE use should be restricted to acute settings such as in CD. MRE has been found to be highly accurate for identifying UC activity and severity, but its adoption in real-life clinical activity, as what has already been reported according to CD, is limited by its long duration, expensiveness, reduced availability and poor tolerability, since bowel preparation and intravenous contrast agents are needed.

### 3.2. Diffusion Weighted Imaging

#### 3.2.1. Assessing Disease Activity

Very recently, DWI-MR has emerged as a highly accurate tool in detecting colonic inflammation in UC [31]. An observational study, carried out on the accuracy of DWI-MR imaging for assessing disease activity in 35 UC patients without oral/rectal preparation and fasting demonstrated that the endoscopic activity was accurately identified by a segmental Nancy score > 1 (sensitivity 89.4%, specificity 86.7%, AUROC, 0.92; *p* = 0.0001) [31]. The segmental and total Nancy scores agreed with the segmental and total modified endoscopic Baron scores (*r* = 0.659, *p* < 0.0001; and *r* = 0.813, *p* = 0.0001, respectively) [31]. DWI hyperintensity independently predicted endoscopic activity (OR 13.26, AUROC, 0.854; *p* = 0.0001) [31]; additionally, DWI hyperintensity had the same accuracy as gadolinium-based contrast agent enhancement in detecting endoscopic inflammation [31]. These results were subsequently confirmed by a later study by Yu et al., which showed that performing DWI at a *b* value of 800 s/mm^2^ enhanced the accuracy of DWI hyperintensity in detecting endoscopic inflammation (sensitivity and specificity were 93% and 79.3%, respectively; AUC of 0.867, *p* < 0.0001) [80]. The *b* value is a DWI parameter that quantifies the level of diffusion weighting applied and it derives from gradient amplitude, duration and interval between paired gradient pulses; it can assume values from 0 to 1000 s/mm^2^. Furthermore, the authors found a threshold ADC value of 2.18 × 10^−3^ mm^2^/s reliably detected endoscopic inflammation (sensitivity and specificity were 89.7% and 80.3%, respectively [80].

In summary, DWI, using the Nancy score, has been shown as a highly valuable, quick and accurate option for assessing disease activity in UC patients.

#### 3.2.2. Monitoring of Disease

A recent study on a UC cohort evaluated the accuracy of the DWI Nancy score in assessing MH, defined by a Mayo endoscopic subscore of 0 to 1, and the treatment response in a subgroup of subjects with active UC. MH was reliably detected by a Nancy score < 7 (sensitivity 75%, specificity 67%, AUROC 0.72; *p* = 0.0063) [81]. The Nancy score has been shown to have good reliability (ICC 0.63) [81]. In those patients achieving MH, both the Nancy score and the Mayo endoscopic subscore significantly decreased (18.2 at baseline vs. 3 at revaluation for Nancy score, *p* = 0.006; 2.4 at baseline vs. 0.6 at revaluation for Mayo endoscopic subscore, *p* = 0.02) [81]. Conversely, the Nancy score did not have significant changes in active patients at revaluations [81].

Overall, these findings underline that the Nancy score is simple, reproducible and sensitive to therapeutic changes. DWI is a fast, easy, acceptable and accurate option for monitoring therapeutic response in UC. Its implementation in clinical practice is hampered by the same drawbacks explained above for CD.

### 3.3. Bowel Ultrasound

#### 3.3.1. Assessing Disease Activity

Even if less investigated than in CD, recent data support the role of BUS in the management of UC patients. BUS has great accuracy in detecting colonic inflammation in UC (sensitivity and specificity of 90% and 96% per-patient analysis, and 74% and 93% per segment analysis, respectively) as endorsed by meta-analysis [18].

The correlation between BWT detected at BUS and the Mayo endoscopic subscore had been previously reported by several retrospective studies (*p* < 0.0001, respectively) [82].

Due to the need of objective, reproducible and comparable measures of inflammation relying upon BUS assessment, Allocca et al. conducted a study to identify reliable BUS parameters of disease activity, enrolling 53 UC patients who underwent BUS and CS within a week [83]. Colonic wall thickness (CWT) (per 1 mm increase: OR, 4.05; *p* = 0.01) and colonic wall flow (CWF) (OR, 7.99; *p* = 0.09) were proved as independent predictors for endoscopic activity (defined by Mayo endoscopic subscore > 2) according to the multivariable analysis [83]. Both CWT and CWF were used to develop Humanitas Ultrasound Criteria (HUC) for detecting disease activity: (a) the presence of a CWF, and CWT > 3 mm; (b) the absence of a CWF, and CWT > 4.43 mm. They both were highly accurate for the identification of disease activity (sensitivity 71% and specificity 100%; AUROC 0.891) [83]. Recently, an external validation study of HUC, now called Milan Ultrasound Criteria (MUC), was provided in an independent cohort of 43 UC patients [84]. The coefficients of CWT and CWF (i.e., 1.4 and 2.0, respectively), obtained from the multivariable analysis in the derivation study, were adopted to calculate MUC (i.e., MUC = (1.4 × CWT) + (2.0 × CWF)) [84]. The MUC significantly agreed with the Mayo endoscopic subscore (*r* = 0.76; *p* < 0.0001) [84]. In addition, as found in the derivation study, authors reported that a MUC score > 6.2 was the most reliable threshold to detect endoscopic activity (sensitivity 85%, specificity 95%; AUROC 0.902) [84].

Finally, since visualization of the rectum is very difficult through transabdominal US, TPUS has been proposed as a potentially more accurate tool in this setting. A recent study found that rectal BWT evaluated through TPUS was correlated with endoscopic and histological scores and was more predictive of endoscopic activity than BWT assessed by transabdominal US (sensitivity 100%, specificity 45.8%, *p* = 0.0002) [85]. These findings are highly valuable in UC since disease activity in the rectum is often the most severe and clinically relevant; TPUS may reliably assess pouchitis, too.

In summary, BUS can accurately assess disease activity in UC. MUC, currently the only US-based validated score in UC, is a reproducible, easy, noninvasive and reliable tool for detecting and grading disease activity in UC patients.

Table 2 shows the accuracy of CTE, MRE, DWI, and BUS and their strengths and weaknesses in assessing UC activity.

#### 3.3.2. Predicting Outcomes and Monitoring

As concerns the ability of BUS to predict clinical outcomes, Parente et al. found that a severe BUS score (BWT > 6 mm and the presence of BWF) after three months of steroid therapy was predictive of a severe endoscopic activity at 15 months (OR 9.1) [86].

Furthermore, MUC was shown to have a predictive value on UC course in both the short and the long period [87]. Indeed, a very recent study on UC patients, investigating BUS assessments in a follow-up period of at least one year, identified a MUC > 6.2 as an independent predictor of need for treatment escalation at 12 months (OR 5.95; *p* < 0.020) [87]. Analysis of the long-term follow-up found that patients with a MUC > 6.2 had a higher risk of need for hospitalization and surgery in comparison to those with MUC < 6.2 (*p* = 0.046; *p* = 0.023, respectively) [87]. Further studies have reported a good agreement between BWT and disease activity in UC patients with active disease undergoing BUS both at baseline and after two months from the initiation of steroid therapy [88]. The authors observed a significant reduction in BWT in patients with clinical improvement (7.3 mm vs. 5.0 mm; *p* < 0.001), but not in those experiencing no clinical improvement (7.0 mm vs. 7.7 mm; *p* not significant) [88].

Maaser et al. carried out a longitudinal, multicenter, prospective study (TRUST&UC), recruiting 224 patients with active UC (Short Clinical Colitis Activity (SCCAI) > 5 points) starting therapy, aiming to assess the accuracy of BUS in evaluating therapeutic response [89]. The authors found that both BWT and BWF significantly reduced even at two weeks from baseline (*p* < 0.001) [89]; furthermore, a significant correlation between BWT normalization (<4 mm for sigmoid colon, <3 mm for the other colonic tracts) and clinical response (decrease in SCCAI by > 3 points vs. baseline) was reported at week 12 from baseline (*p* < 0.001) [89].

In summary, BUS has been found to be highly reliable for evaluating therapeutic changes in UC. Furthermore, MUC have been shown as independent predictors of short- and long-term outcomes in UC.

## 4. Discussion

This review clarifies the current evidence on the accuracy of cross-sectional imaging in monitoring IBD patients.

The sensitivity and specificity for assessment of CD activity have been assessed to be 85% and 91% for bowel US, 81% and 88% for CTE and 80% and 82% for MRE [10,18,19,47,48]. All three procedures can identify CD complications with both a sensitivity and a specificity greater than 80% [10]. In CD, MRE and BUS have been found to accurately detect remission, compared with the reference standard of endoscopic MH, in response to therapy (accuracy was 83% and 86.4% for MRE and BUS, respectively) [26,66]. In UC, MRE and BUS have been shown to reliably assess disease activity (sensitivity 87% and specificity 88% for MRE; sensitivity 90% and specificity 96% for BUS) [13,18]. Importantly, BUS can reliably evaluate therapeutic responses in active UC [89]. In addition, DWI can accurately assess IBD activity (sensitivity and specificity higher than 85% for both CD and UC) [30,31] and MH in response to therapy (sensitivity and specificity were 70% and 80%, respectively, for CD, and 75% and 67%, respectively, for UC) [37,81].

As CS allows the direct view of the mucosa and tissue sampling for diagnosis confirmation, it represents the goal standard procedure, being hardly replaceable, for the initial diagnosis of ileo-colonic CD and UC [5,6]. Moreover, as MH has been selected as the primary treatment target in CD and UC, CS has gained further relevance in terms of monitoring tools for assessing MH [3,4,90]. However, practical limitations such as invasiveness, high costs, poor acceptability and potential related adverse events hamper the use of CS for tight and close monitoring required by the treat-to-target strategy [8,9]. Adopting cross-sectional imaging techniques for this purpose can overcome those CS limitations. Additionally, CS is unable to assess either proximal areas of the small bowel or transmural complications, whose evaluation is mandatory in CD [7]. Indeed, CD behavior tends to change over time (45.9% of patients over 10 years had a change), often evolving from a non-stricturing non-penetrating phenotype to either stricturing or penetrating disease [7]. Otherwise, cross-sectional imaging techniques allow simultaneous evaluation of mural and transmural disease burden of both the small and large bowel, which is crucial for optimal CD management.

Furthermore, there is growing evidence that TH may become the future optimal therapeutic target to meet in IBD management, especially in CD [29,63,67,68]. Thus, cross-sectional imaging techniques may assume an even more relevant role in IBD assessment over the next few years. The field where CS will remain unreplaceable is colorectal cancer surveillance in long standing UC and Crohn’s colitis and the diagnosis of cytomegalovirus (CMV) infection in UC [91].

In our view, BUS and other cross-sectional imaging techniques, being highly accurate, cost-effective, less invasive and well-accepted procedures, are more suitable than CS for tight and close monitoring over disease course.

However, use of CTE for frequent monitoring, even if it is a widely available, short-lasting and cheap procedure, is hampered by radiation exposure and need of an intravenous contrast agent and should be reserved for acute cases.

Conversely, MRE is a radiation-free, high quality imaging technique, suitable for regular reassessment of young patients, but it is limited by high costs, low availability, long examination time and need of intravenous gadolinium-based contrast agents.

The implementation of DWI in clinical practice, although it is easier and faster than MRE and neither bowel cleansing nor intravenous contrast agents are routinely required, is instead hindered by sequence heterogeneity as DWI scanners and procedures are not standardized.

**Figure 1:**
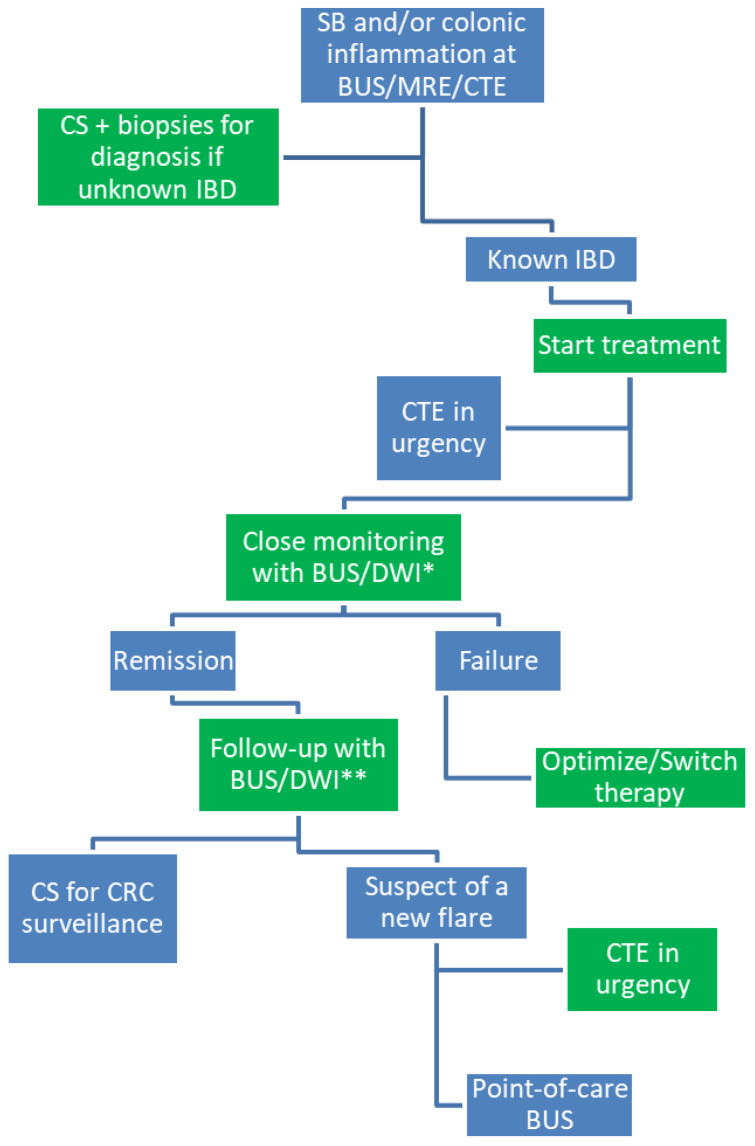
Proposed flowchart for the use of cross-sectional imaging techniques for monitoring of inflammatory bowel disease (IBD). * Every three months for active disease. ** Every six to twelve months in clinical and biochemical remission. BUS, bowel ultrasound; MRE, magnetic resonance enterography; CTE, computed tomography enterography; CS, colonoscopy; DWI, diffusion weighted imaging; CRC, colorectal cancer.

BUS is a short-term, feasible, repeatable, cheap, highly available and tolerable procedure, which can be performed at the point-of-care at the patient’s bedside, having a huge impact on clinical management [65]. Although BUS accuracy and performance have conventionally been thought of as operator-dependent, good to excellent concordance was showed by studies assessing BUS inter-observer variability [92]. Furthermore, the IBUS group is focusing on educational trainings and development of disease activity scores aiming at standardization of procedures [58]. According to this growing evidence reported above, BUS has been found to be as accurate as MRE and CS for assessing disease activity, therapeutic response and predicting short- and long-term outcomes in IBD. Therefore, BUS is rapidly proving as the most usable tool for tight and close monitoring of IBD patients over the disease course (Figure 1). Based on these data, the next step is the incorporation of BUS in real-word management as well as in international clinical trials.

## Figures and Tables

**Table 1:** Accuracy, advantages and disadvantages of the different imaging techniques for CD.

Technique	Sensitivity	Specificity	Strengths	Limitations
CTE	84% for diagnosis [18] 81% for activity [10] >80% for strictures [10] >80% for fistulas and abscesses [10]	95% for diagnosis [18] 88% for activity [10] >80% for strictures [10] >80% for fistulas and abscesses [10]	Low costs, high availability, short examination time	Radiation exposure, intravenous contrast agent, bowel preparation
MRE	93% for diagnosis [18] 80% for activity [10] 89% for strictures [10] 76% for fistulas [10] 86% for abscesses [10]	93% for diagnosis [18] 82% for activity [10] 94% for strictures [10] 96% for fistulas [10] 93% for abscesses [10]	Radiation-free, detailed high-quality imaging	Intravenous contrast agent, bowel preparation, high costs, poor availability, long-lasting procedure
DWI	92.9% for activity [30]	91% for activity [30]	Intravenous contrast agent not required, easier and quicker than MRE, fasting and bowel preparation needed only for SB assessment	Scanners and examinations are heterogeneous Less precise anatomic view than MRE
BUS	85% for diagnosis [18] 85% for activity [10] 79% for strictures [10] 74% for fistulas [10] 84% for abscesses [10]	96% for diagnosis [18] 91% for activity [10] 92% for strictures [10] 95% for fistulas [10] 93% for abscesses [10]	Low costs, radiation free, high availability and acceptability, easy, performed at the point-of-care	Conventionally regarded as operator-dependent

CD: Crohn’s disease; CTE: Computed tomography enterography; MRE: Magnetic resonance enterography; DWI: diffusion-weighted imaging; SB: Small bowel; BUS: Bowel ultrasound.

**Table 2:** Accuracy, advantages and disadvantages of the different imaging techniques for assessment of activity in UC.

Technique	Sensitivity	Specificity	Strengths	Limitations
CTE	74% [81]	>85% [81]	High affordability, short-lasting examination, cheap	Radiation exposure, intravenous contrast agent, bowel cleansing
MRE	87% [13]	88% [13]	No radiation exposure	Intravenous contrast agent, bowel preparation, time-consuming procedure, costly
DWI	89.4% [31]	86.7% [31]	Fast, no radiation exposure, no intravenous contrast agent, no fasting, no bowel preparation	Not standardized DWI scanners and procedures
BUS	90% [18]	96% [18]	No radiation exposure, available, tolerable, cheap, performed at the point-of-care	Traditionally considered operator-dependent

UC: Ulcerative Colitis; CTE: Computed tomography enterography; MRE: Magnetic resonance enterography; DWI: diffusion-weighted imaging; BUS: Bowel ultrasound.
